# Supplementary material for: Barriers to Adopting a Plant-Based Diet in High-Income Countries: A Systematic Review
Source: Nutrients. 2024 Mar 14;16(6):823. doi: 10.3390/nu16060823 (PMC10975979; doi:10.3390/nu16060823)
Supplement: Supplementary file 1 [file nutrients-16-00823-s001.zip › nutrients-2890354-supplementary.pdf]

Supplementary materials

File S1: LSHTM Ethics Application & CARE Form

LSHTM Ethics Application & CARE Form

Project Information

Staff members/students based at:

- (•) LSHTM
- ( ) MRC Gambia@LSHTM (For LSHTM staff/students requiring SCC/MRCG EC review, please select this option)
- ( ) MRC Uganda@LSHTM
- ( ) Other external applicant applying to the SCC/MRCG EC only

1. Full project title

The barriers to adopting a plant-based diet: a systematic review

1a. Basic project information will be made available on the LSHTM website following a favourable ethical opinion. Do you require your project information to be withheld? (see information icon for details on the information that will be made available) (this does not apply to MSc student projects).

- ( ) No
- ( ) Yes
- (•) Not applicable (MSc student)

2. Is this Project in fulfillment of a degree?

(•) Yes ( ) No

2a. Degree registered for

MSc

2e. Indicate student type

- ( ) Distance Learning
- (•) Face-to-Face or Intensive Online

2f(MSc) Is this an original submission, or are you responding to a request for clarification or insufficient information notification from the LSHTM Ethics Committee?

- (•) Original submission
- ( ) Responding to the Ethics Committee

Student Details

3a. Student details

Title	First Name	Surname
[redacted]	[redacted]	[redacted]

Telephone

Email [redacted]

3a(i). Please provide your student ID number

[redacted]

3a(ii). MSc course

Nutrition for Global Health

3a(iii). Indicate proposed project length:

- (•) Standard
- ( ) Extended

3b. Are you studying as a full or part time student?

[redacted]

3b(i). Year of study

[redacted]

3c. Supervisor's name.

[redacted]

3c (i). Supervisor's email address (if more than one, please only provide the email address of your main supervisor)

Email [redacted]

3 c(ii). Supervisor's institution

- (selected) LSHTM
- MRC Gambia or Uganda
- Other

3e. Supervisor status

Confirmed

Project Type

Note: Completing the filter will enable and disable sections of the form so you may not see all questions.

4. Does the research involve primary data collection, analysis of data/samples that have already been collected, or a mix of both?

- Primary
- (selected) Previously collected data/samples
- Mixed

4a(iii). Select type of project:

Literature review/Systematic review only

4c. Does the project involve extraction of data from patient records (e.g. medical, social care, service user records)? (This refers to primary data collection from records and does not include data that was previously collected and is now being used in a secondary analysis).

- Yes
- (selected) No

6. Does this project require review by the Commercialisation and Rapid Response (CaRR) ethics committee? (please see info icon for the remit of this committee)

- Yes
- (selected) No

Samples

6a. Does this research project involve the collection, or use of previously collected, human tissue samples e.g urine, stool, blood etc? (Please select yes even if the samples are not considered relevant material under the Human Tissue Act)

- Yes
- (selected) No

6b. Will this project involve living animals (either laboratory, livestock or wild animals) AND/OR biological material that has been obtained from animals in the experiments planned?

- Yes
- (selected) No

Fast-Track

7a. Will this project be conducted in conjunction with NHS staff, premises or any other connection to the NHS?

- Yes
- (selected) No

7b. Is this application for fast-track? Please see information icon for which projects are eligible. Note: MSc applications are not eligible for fast-track.

- Yes
- (selected) No

Security Sensitive Research Material

9. Does this research involve access to and/or storage of security sensitive research material? Please note that while some data is considered sensitive, such as HIV status, it is not necessarily considered security sensitive. If you are using data that could be considered sensitive, but not security sensitive please answer no to whether your research involves access to and/or storage of security sensitive research material. Please see information icon for what is considered security sensitive material.

- ○ Yes
- ● No

Geography

10. List the countries where the research project is to be conducted (For example: if you are conducting a secondary data analysis for your project and you will be based in the UK, select UK regardless of where the original data has come from):

United Kingdom

Please be aware that all primary health research conducted in the UK requires a sponsor. Please contact the RGIO at RGIO@lshtm.ac.uk for more information on sponsorship.

Outline

Note: Please do not copy and paste directly from the protocol. Applications where large portions of text have been copied and pasted directly from the protocol, and therefore do not properly answer the question, will be invalidated

11. Proposed start date of the project

13/04/2022

11a. Proposed end date of the project

31/08/2022

12. Give a lay outline of the proposed project, including background to the proposal. Include information from any systematic reviews that have been conducted. Sufficient detail must be given to allow the Committee to make an informed decision without reference to other documents, and the outline should be written in such a way that lay members of the committee can make an informed decision.

For this review, I will firstly search the databases (Medline, Embase, Global Health and Web of Science) using my search strategy of combing the Boolean AND/ OR operators. I will use my search terms of "Plantbased", "Plant based", "Plant based diet**, "Plant based food**, "Plant based eating", "Vegetarian**, "Semi vegetarian**, "Strict vegetarian**, "Flexitarian**, "Pescatarian**, "Ovo- vegetarian**, "Lacto-ovo vegetarian**, "Pollotarian**, "Vegetable-based", "Vegetable-based diet**, "Vegan**, "Low-meat diet**,"Meatless diet**, "Dairy-free", "Lactose-free", "Sustainable diet**, "Plant forward diet**, "Vegetable forward diet**, "Adult", "Middle age", "Adulthood", "Young adult**, "Years old", "Year old**, "High income country**, "HIC", "Andorra", "Australia", "Belgium" etc. I will most likely use a mixed-methods design as I expect to find both qualitative and quantitative studies. I expect to find qualitative, case-control, cohort studies etc., for the topic I am researching. I will also use grey literature to access government reports, for example, by using search engines such as Google and Open Grey. After compiling my literature, I will conduct a literature analysis. I will start by importing my references onto EndNote. This is where duplicates will be removed. I will then screen each title and abstract for my eligibility criteria using an eligibility criteria form. I can then use Rayyan to categorise the studies into key barriers. Once I have a set of papers that fit into my criteria, I will use a data extraction form to obtain information such as the author's names, the title of the study, date, the type of study, key findings etc. After I have this information, I can begin the write up of my findings and the entire project. For my review, I aim to understand what barriers may be present for individuals trying to adopt a plant-based diet. I want to understand how these barriers may prevent an individual from transitioning to a plant-based diet and how these may be overcome with recommendations.

Plant-based diets have notably increased in countries such as the UK, Canada, Australia, the USA, and Israel. For example, within the UK, the consumption of plant-based food alternatives such as plant-based milk has almost doubled within the past 10 years. The consumption of these foods within the UK increased from 6.7% to 13.1% from 2008-2011 to 2017- 2019. A diet that includes more plant-based foods rather than animal-sourced foods has shown to be linked to positive effects on individuals' health and the environment. However, there is still a need to shift towards a more plant-based diet and substantially reduce the consumption of animal-source foods. This shift could reduce the global environmental and detrimental health effects. This transition can be achieved through policymaker investments and reducing barriers that prevent an individual from adopting a plant-based diet. Therefore, I want to understand what might prevent individuals from moving towards a plant-based diet as evidence has shown the numerous health and environmental benefits.

Consuming a plant-based diet has been linked to certain health benefits. For example, WHO recommends that to eat a nutritious diet, it is best to eat a variety of foods and obtain this mainly from plants rather than animal sources. In addition, some evidence has shown that adopting a plant-based diet can help to reduce diseases such as type II diabetes, cardiovascular disease and certain cancers. These positive effects of a plant-based diet may be linked to the increased fibre intake, variety, antioxidants and less saturated fat.

There are also environmental and sustainable benefits of moving towards a more plant-based diet. Consuming a plant-based diet has been shown to lower an individual's environmental impact. For example, a plant-based diet reduces the production of greenhouse gas emissions which is the leading cause of climate change. Additionally, consuming a plant-based diet can also reduce the numerous pressures and burdens on the global food system. These pressures include land use, water use, biodiversity, and animal welfare. For example, in 2015, two-thirds of greenhouse gas emissions came from land use and agriculture within the global food system worldwide. Issues such as these are caused mainly by the production and consumption of non-plant-based foods such as meat and dairy. Therefore, these pressures could be reduced by switching to a plant-based diet.

There has been an increasing worldwide interest in moving towards a more plant-based diet for the reasons stated above. The overall transition to a plant-based diet is beneficial for a healthier individual and planet. Although there has been an increase in individuals consuming a plant-based diet in high-income countries such as Australia and the UK, this dietary transition is still not proceeding fast enough to meet net-zero targets. For example, this could be due to countries not successfully implementing these net-zero targets into their laws and policies. As well as other factors such as cultural barriers. Some countries that have made pledges for net-zero have a history of high meat and dairy consumption. Furthermore, there are other barriers to adopting a plant-based diet, such as enjoyment of non-plant-based foods, negative health perceptions, issues with accessibility, social acceptance, financial restrictions etc. Global issues such as these still prevent individuals from moving towards this more sustainable, environmentally friendly and healthier diet, which is why I want to conduct my research.

12a. Upload the study protocol (compulsory for staff and doctoral students), including data collection forms, questionnaires and topic guides. Please upload each document separately, ensuring that the date and version number of each document is correct.

Documents

Type	Document Name	File Name	Version Date	Version	Size
Protocol / Proposal	[redacted]		17/03/2022	1	44.1 KB

13. State the intended value of the project, detailing why the topic is of interest or relevance. If this project or a similar one has been done before what is the value of repeating it? Give details of overviews and/or information on the Cochrane database. This area is of increasing importance – please ensure you give a full response.

As stated above, a plant-based diet is considered a more sustainable and environmentally friendly way to eat. As a result, there has been a gradual increase in the number of individuals consuming a plant-based diet, particularly in western Europe in recent years. However, despite the numerous advantages of adopting a plant-based diet, individuals still experience issues as to why they cannot make this adjustment. Therefore, I want to conduct a systematic review to understand some of the barriers as to why individuals may struggle to adopt this more sustainable, environmentally friendly and healthier diet. A comprehensive review could help us understand what barriers individuals may face and how these could be overcome to help more individuals who wish to adopt a plant-based diet.

This study may also have implications for policymaking decisions. Through this research, I will gain an insight into what barriers individuals may be experiencing when trying to adopt a plant-based diet. Some of these barriers can be heavily influenced by individual behaviour change. Therefore, this study could provide valuable evidence of what policymakers can implement to drive individual behaviour change and encourage a plant-based diet. For example, the accessibility to plant-based foods is a barrier. This affects an individual's ability to adopt a plant-based diet. Therefore, if we understand that access to plant-based foods is a major barrier to driving individual behaviour change, policymakers could implement policies such as offering plant-based foods in public sector canteens. Therefore, the results from this study could have implications on policymaking as to what we understand as drivers of behaviour change.

A similar review has been done before; however, that review focused on the facilitators and barriers of adopting a plant-based diet. My study will not focus on how plant-based diets impact chronic diseases. The researchers also used a different definition from mine when discussing plant-based diets. They considered a plant-based diet a vegetarian diet, which is not the definition I will be using. I will be describing a plant-based diet as "dietary patterns that have a greater emphasis on food derived from plants (such as fruits and vegetables, whole grains, pulses, nuts, seeds and oils).". The previous review also doesn't have a date range for the study's criteria—some studies dated back to 1974. I will be having a date limit for the studies, going back no further than the year 2000. The barriers individuals may have experienced from 1974 are likely to differ between 50 years ago and today. Therefore, I will be including more relevant and newer studies compared to this previous review. Furthermore, there were no limits for the countries used, and there was a particular focus on Hungarian studies. My study will include only high-income countries.

When completing an initial literature scope before completing the concept note, there were no previous research studies on the Cochrane database discussing barriers to adopting a plant-based diet. When looking further at the Cochrane database, there were no systematic reviews on the Cochrane database related to my research topic. Moreover, the review mentioned above was also not on the Cochrane database.

14. Hypothesis statement.

There will be a range of barriers that individuals experience when adopting a plant-based diet, and these will vary depending on the setting and population.

A potential research question: "What barriers do adults aged 18 to 65 years old from high-income countries experience when trying to adopt a plant-based diet?"

15. Overall aim of project

The overall aim is to systematically understand the barriers an individual may experience when trying to adopt a plant-based diet.

16. Specific objectives of project

To identify key gaps in the evidence and to synthesise this data.
To categorise the evidence into subgroups by using a conceptual map.
To formulate recommendations to overcome the barriers presented in the research.

Methods

Note: Please do not copy and paste directly from the protocol. Applications where large portions of text have been copied and pasted directly from the protocol, and therefore do not properly answer the question, will be invalidated

18. Specify the procedures/methodology to be conducted during the project. Please include outcome measures and plans for data management and analysis. For literature reviews, include details on search strategy, search terms, inclusion and exclusion criteria.

Due to my project type, I will be using the Boolean operators of OR and AND as my search strategy. I will be using multiple search engines such as Medline, Embase, Global Health and Web of Science. I will combine the key search terms: "Plantbased", "Plant based", "Plant based diet*", "Plant based food*", "Plant based eating", "Vegetarian*", "Semi vegetarian*", "Strict vegetarian*", "Flexitarian*", "Pescatarian*", "Ovo- vegetarian*", "Lacto-ovo vegetarian*", "Pollotarian*", "Vegetable-based", "Vegetable-based diet*", "Vegan*", "Low-meat diet*","Meatless diet*", "Dairy-free", "Lactose-free", "Sustainable diet*", "Plant forward diet*", "Vegetable forward diet*", "Adult", "Middle age", "Adulthood", "Young adult*", "Years old", "Year old*", "High income country*", "HIC", "Andorra", "Australia", "Belgium" etc. in the variation of AND and OR. I will adapt my search strategy per search engine. For example, I will use MeSH keywords will be used for Medline. My inclusion criteria will be human studies, studies set in high-income countries, studies that include a barrier to adopting a plant-based diet, and they will need to include my search terms as stated above. The population will be adults aged 18- 65 years. I will most likely use a mixed-methods design as I expect to find both qualitative and quantitative studies. I expect to find qualitative, case-control, cohort studies etc., for the topic I am researching. Therefore, all study designs will be considered. I will also search the grey literature to include government, committee and policy reports, etc. I will use search engines such as Google and Open Grey. I am likely to find very current information on this evolving topic by using grey literature. It will allow me to hear the experiences and opinions from a more diverse audience. I will narrow down the studies obtained using my exclusion criteria. These will be studies set in low-income and middle-income countries, studies published before the year 2000, child studies and studies focusing on only meat alternatives, as well as publications that are reviews and not from the original publication source, such as a systematic review. They will also need to be written in English. And I will exclude studies that include individuals who have diabetes, cardiovascular disease, high blood pressure and obesity. Once all the data is extracted, I will export the searches into EndNote to remove duplicates and begin data synthesis. I will screen each title and abstract using my eligibility criteria form. Once I have the studies that fit into my criteria, I will then extract the data from these using my data extraction form to obtain the required information about the study, such as title, date, key findings etc. I will also use Rayyan as a tool to screen and categorise the papers. The PRISMA checklist will be used, and a flow chart will also be created to show my selection methodology. Furthermore, I will create a conceptual mapping diagram to establish the main themes from my literature analysis. This mapping will help form groups and subgroups to present the data clearly whilst forming coherent conclusions and assessing the barriers' strength.

23. In terms of the feasibility of your project, what could stop this project from succeeding, or prevent you from achieving your objectives? *Please indicate any aspects of your proposed approach which could potentially experience difficulties, e.g. delays with permissions, data collection or storage problems, lack of sufficient comparable information, etc. You may also wish to mention any wider matters which could affect your project, e.g. civil unrest, natural disasters, transport availability.

Overall, there are a few risks for my project, the main issues that may arise include time management concerns and having too few or too many papers. Firstly, due to the type of project I will be conducting, I will have to read countless research papers. I will then have to ensure these fit my inclusion and exclusion criteria and remove any papers that do not. I will also have to remove any duplicate papers, as I will be accessing these from multiple search engines with the same papers. This will be a very time-consuming process and will likely take more time than expected. As I need to ensure I will have enough time for the literature analysis as well as the write up of the entire project. During this stage or near the end, I may realise I have too many or too few papers.

Secondly, having too few papers may be a problem that stops my project from succeeding or me from achieving my objectives. For example, I did an initial literature scope when I first developed my project idea and completed my concept note. I used several search engines, and there seemed to be an adequate number of research papers for me to complete my review. However, this was without properly considering my inclusion and exclusion criteria during the initial project development phase. Therefore, I may run into the problem of not having enough research papers in order to achieve my objectives.

Lastly, I could have a problem with having too many studies. It is more likely that I will have too many studies than too few due to the nature of my project. However, due to the vastness of my project topic, I hope to gain a varied insight and understanding of numerous barriers whilst also answering my objectives and overall aim successfully.

24. What alternative plans do you have in case you encounter any of the potential problems you have identified? Please troubleshoot all points raised in question 25.

My project will be time-consuming. I expect reading and analysing the literature will take more time than initially expected. Therefore, I have created a timeline for my entire project. This timeline factors in details from gathering the literature and data analysis to the write-up and submission of my final project. I have then sent this timeline to my supervisor to ensure they are aware of my timeframe and plan for the year. With this plan in place, I will have a structure for my project which can reduce some of the pressures of having a short completion time frame.

With too few papers, I could potentially broaden my criteria to allow for more studies. I could also increase my search terms. However, as I will be using numerous search terms in my search strategy, I am hoping this will allow for many research papers. It is more likely that I will have too many papers than too few.

If I have too many papers, I can alter my criteria and search strategy to be narrower. Furthermore, I can ensure I do not use grey literature, and the studies I use are of a highly rigorous scientific standard.

25. What specific facilities or resources will you personally expect to make use of for your project (eg a local university library, lab facilities, project placement with a specific organisation etc)?

I will use the university's library to work in a quiet space to read papers and write my project. However, apart from this, I will not likely need any other facilities as listed above due to the type of project I will be conducting. I will be using publicly available databases to search for published and grey literature.

27. List key references (no more than 5), including for methods to be used.

Bramer, W. and Bain, P., 2017. Updating search strategies for systematic reviews using EndNote. Journal of the Medical Library Association, 105(3), pp.285-289.

Fehér, András & Gazdecki, Michał & Véha, Miklós & Szakály, Márk & Szakály, Zoltán., 2020. A Comprehensive Review of the Benefits of and the Barriers to the Switch to a Plant-Based Diet. Sustainability, 12(10), pp.4136.

Higgins, J., Thomas, J., Chandler, J., Cumpston, M., Li, T., Page, M. and Welch, V., 2021. Cochrane Handbook for Systematic Reviews of Interventions version 6.2. [online] Training.cochrane.org. Available at: https://training.cochrane.org/handbook/current [Accessed 19 December 2021].

Library.leeds.ac.uk. 2021. Literature searching explained. [online] Available at: <https://library.leeds.ac.uk/info/1404/literature_searching/14/literature_searching_explained/4#b oolean%20logic> [Accessed 22 December 2021].

Nutrition.org.uk.2019. Plant-based diets- British Nutrition Foundation. [online] Available at: https://www.nutrition.org.uk/putting-it-into-practice/plant-based-diets/plant-based-diets/ [Accessed 20 December 2021].

Experience

30. State the personal experience of the applicant and of senior collaborators in the research project in the field concerned, and their contribution to this project. Indicate any previous work done related to the project topic including student and/or professional work, or publications

I have previously completed comprehensive literature reviews as part of my background for multiple research studies, essays, and undergraduate dissertation. I have also completed the John Hopkins short course on Introduction to Systematic Review. Although I have no previous academic experience with the topic of interest. However, my supervisor's research links to diets, the environment and human health. [redacted]

30a. Upload the CVs for all main investigators working on the project. For MSc students, please upload your CV only.

Documents

Type	Document Name	File Name	Version Date	Version	Size
Investigator CV	[redacted]		16/03/2022	1	28.1 KB

30e. Have the main investigators undertaken any Research Ethics/Human Subjects Protection training (either online or face to face)? It is recommended that all applicants and members of their research team should complete ethics training at least every 3 years to account for changes in guidelines and regulations. Links to online training can be found in the information icon. (Please note this is not the same as GCP training).

(•) Yes
( ) No

30e(i). Please upload a copy of the certificate(s)

Documents

Type	Document Name	File Name	Version Date	Version	Size
Other	Research_Ethics_online_training_certificate	Research_Ethics_online_training_certificate.pdf	02/02/2022	1	164.9 KB

Confidentiality & Data

40. State how your data will be stored and what will be done with it at the end of the project.

As my project is a systematic review, all data I will be accessing is of public domain. Therefore, anyone can access this information. The papers will be exported into EndNote, where I will delete repeats, ensure they are part of my criteria and analyse the data. I will also be using Rayyan to help categorise and analyse the data. This data is not sensitive as it is sourced from public domain sites. After submitting my project, I will keep my EndNote and Rayyan databases for 3 years. After the 3 years, I will delete the EndNote and Rayyan data.

Funding

46. Do you have external funding for this project?

( ) Yes
(•) No

46b. How will the project go ahead without funds?

Funding will not be needed for my project, as it will be own funded as part of my MSc.

46c. Are you in receipt of any funding from the United States? Or will you be collaborating with (or with individuals from) a US Institution/organisation?

( ) Yes
(•) No

47. Has the project been sent out for peer/independent scientific review (please select yes if the project is being sent to the SCC)?

(•) Yes
( ) No

47b. If yes, who has provided peer/independent scientific review of the project?

A concept note was completed for my project and sent out for review. It was then reviewed by my supervisor as well as another independent staff member. I then incorporated their feedback to further my project development and to include it within this CARE form.

49. Does the Chief Investigator or any other investigator/collaborator have any direct personal involvement (e.g. financial, share holding, personal relationship etc.) in the organisations sponsoring or funding the research that may give rise to a possible conflict of interest?

( ) Yes
(•) No

50. Will individual researchers receive any personal payment over and above normal salary, or any other benefits or incentives, for taking part in this research?

- ○ Yes
- ◉ No

Data Sources, Results & Permissions

70. If you expect to use existing data, how will you obtain it? *Indicate who holds the data, who specifically you will contact, and by when. Any contact so far, especially anything confirmed in writing, should be mentioned.

I will be using public domain data.

70a. Please upload any documents confirming that you have requested/been granted permission to use any existing data. This can include email correspondence as well as formal letters of permission. (This is mandatory for projects using existing data).

71. If you expect to use any public domain data, please give further details. *Make clear how you will gain access. Public domain data must be freely available to any member of the public, without any restrictions or requirement for special permission, and must not enable the identification of living people.

For my type of project, I will be using public domain data. If necessary, I will create an account for the search engines to have full access to all the research papers. All the papers that I will be using are already published and accessible.

71a. Please provide the links to all public domain data that you expect to use.

https://ovidsp.dc1.ovid.com/ovid-b/ovidweb.cgi?
ID=shib%3Adc1%3A0xb46ee6c435bd495198d9173bd308bbfc&PASSWORD=0xb46ee6c435bd495198d9173bd308bbfc&idpselect=https%3A%2F%2Fidp.lshtm.ac.uk%2Fshibboleth&CSC=Y&T=JS&D=medall&NEWS=n&entityID=https%3A%2F%2Fidp.lshtm.ac.uk%2Fshibboleth&PAGE=ma

https://ovidsp.dc1.ovid.com/ovid-b/ovidweb.cgi?
ID=shib%3Adc1%3A0x05be0fb90f624f2a84957c2b85e18b78&PASSWORD=0x05be0fb90f624f2a84957c2b85e18b78&idpselect=https%3A%2F%2Fidp.lshtm.ac.uk%2Fshibboleth&CSC=Y&T=JS&D=emczd&NEWS=n&entityID=https%3A%2F%2Fidp.lshtm.ac.uk%2Fshibboleth&PAGE=main

https://ovidsp.dc1.ovid.com/ovid-b/ovidweb.cgi?
ID=shib%3Adc1%3A0x37ef51ee49254e18a74db951c14071d4&PASSWORD=0x37ef51ee49254e18a74db951c14071d4&idpselect=https%3A%2F%2Fidp.lshtm.ac.uk%2Fshibboleth&CSC=Y&T=JS&D=cagz&NEWS=n&entityID=https%3A%2F%2Fidp.lshtm.ac.uk%2Fshibboleth&PAGE=mai

https://www.webofscience.com/wos/woscc/basic-search

72. Will any specific data rights permissions or usage limitations be required regarding data to be used or collected in the project?

- ○ Yes
- ◉ No

73. Will any agreements be required regarding data to be used or collected in the project (for example, material transfer agreements or data transfer agreements)?

- ○ Yes
- ◉ No

74. Are there any existing obligations regarding ownership of results to third parties (e.g. employer)?

- ○ Yes
- ◉ No

Type of risk

75. Where will the project be carried out? *Note that work away from LSHTM, your primary residence, or outside the UK means any form of work for your project, not just primary data collection. Some courses may have specific restrictions on this.

All work will take place either at LSHTM or my primary residence

76. Please indicate which locations you expect to work at. Check all that apply.

- ☐ Field Work
- ☑ Desk-based (Home, LSHTM, Office, Library)
- ☐ Lab Work
- ☐ Other work away from home

77. Will the project involve working with or handling any of the following materials?

- ☐ Pathogenic organisms
- ☐ Human tissue
- ☐ Animal tissue
- ☐ Radiochemicals
- ☐ Genetically modified organisms
- ☐ Toxic chemicals
- ☑ None of the above

78. Are any other potentially hazardous activities likely to be carried out during the project?

- ○ Yes
- ◉ No

Well-being

111. If you will be conducting research away from the School, and have a disability that may require support, you are encouraged to contact Studentadvice@lshtm.ac.uk in good time to discuss support options.

- [x] I have read and understood the above information

112. Your wellbeing is of paramount importance and if your project research will mean spending time away from usual support networks, and if you think that you could find this challenging, we strongly encourage you to seek support and advice well in advance. You can book a confidential appointment (link to appointment booking form available in the information icon) with one of the Student Counsellors to discuss any concerns. The Counselling Service is available to all students and you do not have to have a diagnosed condition to seek support.

- [x] I have read and understood the above information

Signature Instructions

The form should be completed and finalised prior to signing or requesting signatures. Incomplete or incorrectly filled in forms will be invalidated and this may cause a delay with your review. For external applicants/supervisors, please ensure that they have registered for an account prior to requesting the signature.

Please ensure that you obtain all signatures on this page. Failure to obtain all required signatures will result in the form not submitting and a delay to the processing of your application. If you have trouble signing/requesting signatures please contact ethics@lshtm.ac.uk or MScEthics@lshtm.ac.uk (for MSc students).

For MSc students:

- Please indicate that you have read and understood each statement under the student declaration before adding your signature using the blue sign button at the end of the student declaration.
- Once you have signed, please request your supervisor signature using the request signature button under the supervisor signature section. For external supervisors, please ensure that they have registered for an account prior to requesting the signature.
- Once your Supervisor has signed, please request your Course Director/Project Module Organiser signature using the request signature button under the Course Director / Project Module Organiser Signature section.
- Check whether any further signatures are required (these will show under the Course Director / Project Module Organiser Signature section) and request as required.
- Please ensure you have obtained all required signatures are your form will not submit without these. If you have any trouble please contact MScethics@lshtm.ac.uk

Signature - Applicant

Student Declaration

- [x] I have read and understood, and agree to abide by the LSHTM Good Research Practice policy as well as all applicable Standard Operating Procedures, including on informed consent
- [x] I undertake to abide by all regulations, guidelines and standards of good practice, including but not limited to the Data Protection Act 2018, GDPR, and the Declaration of Helsinki
- [x] I undertake to abide by all local rules/laws for non-UK research
- [x] I agree to conduct my project on the basis set out in this form, and to consult staff (initially, my Supervisor) if making any subsequent changes
- [x] I agree to inform the ethics committee of any changes made to this form, and will not implement any changes until approval from the ethics committee has been received
- [x] I undertake to adhere to all conditions set out by review bodies in giving approval and will not start the project until all required approvals are in place
- [x] I agree to comply with the relevant safety requirements, and will submit a separate request for LSHTM travel insurance where relevant
- [x] I agree to inform the Faculty Safety Officer and/or the Off-Site Safety Advisor (as required) if there are any changes to the risk assessment
- [x] I confirm that there are no conflicts of interest that preclude my participation in the project

Student signature

Signed: This form was signed by [redacted] on 12/04/2022 10:12

Signature - Supervisor

Supervisor signature

I declare that:

- I agree that the information submitted in this application is a reasonable summary of the proposed project.
- I agree that this form correctly indicates whether or not ethics approval will be required.
- I agree that this form contains adequate information for the ethics committee to form an opinion of the proposed project.
- I agree that all required supporting documentation is attached to this application.
- (For MSc projects only) I agree that responses in the Risk Assessment section address the main risks connected with a project of this nature
- I have reviewed the risk of the project, including travel, and agree that it is an acceptable risk to the student
- I confirm that there are no conflicts of interest that preclude my role as supervisor for this project
- I Have read and understood, and agree to abide by the LSHTM Good Research Practice policy

Signed: This form was signed by [redacted] on 12/04/2022 18:06

Signature - Course Director/Project Module Organiser

Course Director / Project Module Organiser Signature

I declare that:

- I agree that the proposed project's academic content is suitable for this MSc

Signed: This form was signed by Dr. Marko Kerac (marko.kerac@lshtm.ac.uk) on 20/04/2022 14:36

Signature - Other

Note:

The form will automatically submit upon receipt of all required signatures.

After submission, you will receive a confirmation email with further details.

If you have not received a confirmation email within 5 working days please email ethics@lshtm.ac.uk (staff) or MScethics@lshtm.ac.uk (students) to check the status of your submission.

File S2: Ethical approval letter

London School of Hygiene & Tropical Medicine
Keppel Street, London WC1E 7HT
United Kingdom
Switchboard: +44 (0)20 7636 8636

www.lshtm.ac.uk

MSc Research Ethics Committee

[redacted]
MSc Student
Nutrition for Global Health
LSHTM

22 April 2022

[redacted]

Study Title: The barriers to adopting a plant-based diet: a systematic review

LSHTM MSc Ethics ref: 26908

Thank you for submitting your application for the above MSc research project.

As your projectis a systematic/literature review only, it was assessed by the Research Governance & Integrity Office as not requiring ethical approval from the MSc ethics committee. It is the student's responsibility to ensure that all other required approvals are in place before starting the research project.

Any subsequent changes to the application must be submitted to the Committee via an Amendment form on the ethics online applications website: http://leo.lshtm.ac.uk .

Best of luck with your project.

Yours sincerely,

Rebecca Carter

Ethics Facilitator

MScEthics@lshtm.ac.uk
http://www.lshtm.ac.uk/ethics/

Improving health worldwide

File S3: PRISMA checklist

Section and Topic	Item #	Checklist item	Location where item is reported
**TITLE**			
Title	1	Identify the report as a systematic review.	1
**ABSTRACT**			
Abstract	2	See the PRISMA 2020 for Abstracts checklist.	1
**INTRODUCTION**			
Rationale	3	Describe the rationale for the review in the context of existing knowledge.	1-3
Objectives	4	Provide an explicit statement of the objective(s) or question(s) the review addresses.	3
**METHODS**			
Eligibility criteria	5	Specify the inclusion and exclusion criteria for the review and how studies were grouped for the syntheses.	3-4
Information sources	6	Specify all databases, registers, websites, organisations, reference lists and other sources searched or consulted to identify studies. Specify the date when each source was last searched or consulted.	3-6
Search strategy	7	Present the full search strategies for all databases, registers and websites, including any filters and limits used.	3-6<br>File S4
Selection process	8	Specify the methods used to decide whether a study met the inclusion criteria of the review, including how many reviewers screened each record and each report retrieved, whether they worked independently, and if applicable, details of automation tools used in the process.	4-6
Data collection process	9	Specify the methods used to collect data from reports, including how many reviewers collected data from each report, whether they worked independently, any processes for obtaining or confirming data from study investigators, and if applicable, details of automation tools used in the process.	4-6
Data items	10a	List and define all outcomes for which data were sought. Specify whether all results that were compatible with each outcome domain in each study were sought (e.g. for all measures, time points, analyses), and if not, the methods used to decide which results to collect.	3-5
	10b	List and define all other variables for which data were sought (e.g. participant and intervention characteristics, funding sources). Describe any assumptions made about any missing or unclear information.	3-5

Section and Topic	Item #	Checklist item	Location where item is reported
Study risk of bias assessment	11	Specify the methods used to assess risk of bias in the included studies, including details of the tool(s) used, how many reviewers assessed each study and whether they worked independently, and if applicable, details of automation tools used in the process.	4-5
Effect measures	12	Specify for each outcome the effect measure(s) (e.g. risk ratio, mean difference) used in the synthesis or presentation of results.	4
Synthesis methods	13a	Describe the processes used to decide which studies were eligible for each synthesis (e.g. tabulating the study intervention characteristics and comparing against the planned groups for each synthesis (item #5)).	4
	13b	Describe any methods required to prepare the data for presentation or synthesis, such as handling of missing summary statistics, or data conversions.	4
	13c	Describe any methods used to tabulate or visually display results of individual studies and syntheses.	4
	13d	Describe any methods used to synthesize results and provide a rationale for the choice(s). If meta-analysis was performed, describe the model(s), method(s) to identify the presence and extent of statistical heterogeneity, and software package(s) used.	4
	13e	Describe any methods used to explore possible causes of heterogeneity among study results (e.g. subgroup analysis, meta-regression).	n/a
	13f	Describe any sensitivity analyses conducted to assess robustness of the synthesized results.	n/a
Reporting bias assessment	14	Describe any methods used to assess risk of bias due to missing results in a synthesis (arising from reporting biases).	4-5
Certainty assessment	15	Describe any methods used to assess certainty (or confidence) in the body of evidence for an outcome.	4-5
**RESULTS**			
Study selection	16a	Describe the results of the search and selection process, from the number of records identified in the search to the number of studies included in the review, ideally using a flow diagram.	5-6
	16b	Cite studies that might appear to meet the inclusion criteria, but which were excluded, and explain why they were excluded.	5-6
Study characteristics	17	Cite each included study and present its characteristics.	5-8

Section and Topic	Item #	Checklist item	Location where item is reported
Risk of bias in studies	18	Present assessments of risk of bias for each included study.	15-16
Results of individual studies	19	For all outcomes, present, for each study: (a) summary statistics for each group (where appropriate) and (b) an effect estimate and its precision (e.g. confidence/credible interval), ideally using structured tables or plots.	7-8<br>Table 1/ File S8
Results of syntheses	20a	For each synthesis, briefly summarise the characteristics and risk of bias among contributing studies.	7-17
	20b	Present results of all statistical syntheses conducted. If meta-analysis was done, present for each the summary estimate and its precision (e.g. confidence/credible interval) and measures of statistical heterogeneity. If comparing groups, describe the direction of the effect.	9-17
	20c	Present results of all investigations of possible causes of heterogeneity among study results.	n/a
	20d	Present results of all sensitivity analyses conducted to assess the robustness of the synthesized results.	n/a
Reporting biases	21	Present assessments of risk of bias due to missing results (arising from reporting biases) for each synthesis assessed.	15-16
Certainty of evidence	22	Present assessments of certainty (or confidence) in the body of evidence for each outcome assessed.	16-17
**DISCUSSION**			
Discussion	23a	Provide a general interpretation of the results in the context of other evidence.	17-19
	23b	Discuss any limitations of the evidence included in the review.	20
	23c	Discuss any limitations of the review processes used.	20
	23d	Discuss implications of the results for practice, policy, and future research.	20-22
**OTHER INFORMATION**			
Registration and protocol	24a	Provide registration information for the review, including register name and registration number, or state that the review was not registered.	The review was not registered
	24b	Indicate where the review protocol can be accessed, or state that a protocol was not prepared.	A protocol was not prepared

Section and Topic	Item #	Checklist item	Location where item is reported
			with PROSPERO
	24c	Describe and explain any amendments to information provided at registration or in the protocol.	n/a
Support	25	Describe sources of financial or non-financial support for the review, and the role of the funders or sponsors in the review.	n/a
Competing interests	26	Declare any competing interests of review authors.	n/a
Availability of data, code and other materials	27	Report which of the following are publicly available and where they can be found: template data collection forms; data extracted from included studies; data used for all analyses; analytic code; any other materials used in the review.	Supplementary materials

File S4: Search strategy

Embase: Result: 1462

(plantbased or plant-based or plant-based diet* or plant-based food* or vegetarian* or vegetarianism or semi-vegetarian* or strict vegetarian* or vegetarian diet* or diet*, vegetarian or flexitarian* or flexitarian diet* or pescatarian* or pescatarian diet* or ovo-vegetarian* or ovo-vegetarian diet* or ovovegetarian diet* or lacto-ovo vegetarian* or lacto-ovo vegetarian diet* or lactovegetarian diet* or pollotarian* or pollotarain diet* or vegetable-based or vegetable-based diet* or vegan* or vegan diet* or diet*, vegan or veganism or low meat diet* or meatless diet* or dairy-free or dairy-free diet* or lactose-free or lactose-free diet* or sustainable diet* or plant-forward diet* or vegetable-forward diet* or vegetarian eating pattern* or meat reduction or low meat or low meat diet* or plant-eating or meat consumption or plant consumption or dairy consumption or veggie or veggie diet* or non-dairy or plant-derived or sustainable food*) AND (adult* or middle age* or adulthood or adult population or young adult* or years old or year old*) AND ((((((((((high-income country* or high-income setting* or HIC or affluent country* or america or andorra or antigua) and barbuda) or aruba or australia or austria or bahamas or bahrain or barbados or belgium or bermuda or british virgin islands or brunei or brunei darussalam or canada or cayman islands or channel islands or chile or croatia or curacao or cyprus or czech republic or denmark or england or estonia or faroe islands or finland or france or french polynesia or germany or gibraltar or greece or greenland or guam or hong kong or hungary or iceland or ireland or isle of man or israel or italy or japan or korea or republic of china or republic or korea or kuwait or latvia or liechtenstein or lithuania or luxembourg or macao or malta or monaco or nauru or netherlands or new caledonia or new zealand or northern mariana islands or norway or oman or palau or poland or portugal or puerto rico or qatar or saint kitts) and nevis) or san marino or saudi arabia or scotland or seychelles or singapore or sint maarten or slovakia or slovak republic or slovenia or spain or st kitts) and nevis) or st martin or states or sweden or switzerland or taiwan or trinidad) and tobago) or turks) and caicos islands) or united arab emirates or united kingdom or UK or united states or united states of america or USA or US or uruguay or virgin islands or wales)

Medline: Results: 3161

(plantbased or plant-based or plant-based diet* or plant-based food* or diets, plant-based or plant-based nutrition or nutrition, plant-based or vegetarian* or diets, vegetarian or vegetarian diet* or semi-vegetarian* or strict vegetarian* or flexitarian* or flexitarian diet* or pescatarian* or pescatarian diet* or ovo-vegetarian* or ovo-vegetarian diet* or lacto-ovo vegetarian* or lacto-ovo vegetarian diet* or vegetarianism or diet*, lacto-ovo vegetarian or lacto- vegetarian diet* or diets, lacto-vegetarian or vegetarian diet*, lacto-ovo or pollotarian* or pollotarain diet* or vegetable-based or vegetable-based diet* or vegan* or vegan diet* or veganism or low meat diet* or meatless diet* or dairy-free or dairy-free diet* or lactose-free or lactose-free diet* or sustainable diet* or plant-forward diet* or vegetable-forward diet* or meat reduction or low meat or low meat diet* or plant-eating or meat consumption or plant consumption or dairy consumption or veggie or veggie diet* or non-dairy or plant-derived or sustainable food*) AND (adult* or middle age* or adulthood or adult population or young adult* or years old or year old*) AND ((((((((((high-income country* or high-income setting* or HIC or affluent country* or america or andorra or antigua) and barbuda) or aruba or australia or austria or bahamas or bahrain or barbados or belgium or bermuda or british virgin islands or brunei or brunei darussalam or canada or cayman islands or channel islands or chile or croatia or curacao or cyprus or czech republic or denmark or england or estonia or faroe islands or finland or france or french polynesia or germany or gibraltar or greece or greenland or guam or hong kong or hungary or iceland or ireland or isle of man or israel or italy or japan or korea or republic of china or republic or korea or kuwait or latvia or liechtenstein or lithuania or luxembourg or macao or malta or monaco or nauru or

netherlands or new caledonia or new zealand or northern mariana islands or norway or oman or palau or poland or portugal or puerto rico or qatar or saint kitts) and nevis) or san marino or saudi arabia or scotland or seychelles or singapore or sint maarten or slovakia or slovak republic or slovenia or spain or st kitts) and nevis) or st martin or states or sweden or switzerland or taiwan or trinidad) and tobago) or turks) and caicos islands) or united arab emirates or united kingdom or UK or united states or united states of america or USA or US or uruguay or virgin islands or wales)

Global Health: Results: 550

(plantbased or plant-based or plant-based diet* or plant-based food* or vegetarian* or vegetarianism or semi-vegetarian* or strict vegetarian* or vegetarian diet* or flexitarian* or flexitarian diet* or pescatarian* or pescatarian diet* or ovo-vegetarian* or ovo-vegetarian diet* or lacto-ovo vegetarian* or lacto-ovo vegetarian diet* or pollotarian* or pollotarain diet* or vegetable-based or vegetable-based diet* or vegan* or vegan diet* or veganism or low meat diet* or meatless diet* or dairy-free or dairy-free diet* or lactose-free or lactose-free diet* or sustainable diet* or plant-forward diet* or vegetable-forward diet* or meat reduction or low meat or low meat diet* or plant-eating or meat consumption or plant consumption or dairy consumption or veggie or veggie diet* or non-dairy or plant-derived or sustainable food*) AND (adult* or middle age* or adulthood or adult population or young adult* or years old or year old*) AND ((((((((((high-income country* or high-income setting* or HIC or affluent country* or america or andorra or antigua) and barbuda) or aruba or australia or austria or bahamas or bahrain or barbados or belgium or bermuda or british virgin islands or brunei or brunei darussalam or canada or cayman islands or channel islands or chile or croatia or curacao or cyprus or czech republic or denmark or england or estonia or faroe islands or finland or france or french polynesia or germany or gibraltar or greece or greenland or guam or hong kong or hungary or iceland or ireland or isle of man or israel or italy or japan or korea or republic of china or republic or korea or kuwait or latvia or liechtenstein or lithuania or luxembourg or macao or malta or monaco or nauru or netherlands or new caledonia or new zealand or northern mariana islands or norway or oman or palau or poland or portugal or puerto rico or qatar or saint kitts) and nevis) or san marino or saudi arabia or scotland or seychelles or singapore or sint maarten or slovakia or slovak republic or slovenia or spain or st kitts) and nevis) or st martin or states or sweden or switzerland or taiwan or trinidad) and tobago) or turks) and caicos islands) or united arab emirates or united kingdom or UK or united states or united states of america or USA or US or uruguay or virgin islands or wales)

Web of Science: Results: 4492

(plantbased OR plant-based OR plant-based diet* OR plant-based food* OR vegetarian* OR vegetarianism OR semi-vegetarian* OR strict vegetarian*OR vegetarian diet* OR flexitarian* OR flexitarian diet* OR pescatarian*OR pescatarian diet* OR ovo-vegetarian* OR ovo-vegetarian diet* OR lacto-ovo vegetarian* OR lacto-ovo vegetarian diet* OR pollotarian* OR pollotarain diet*OR vegetable-based OR vegetable-based diet* OR vegan* OR vegan diet* OR veganism OR low meat diet* OR meatless diet* OR dairy-free OR dairy-free diet* OR lactose-free OR lactose-free diet*OR sustainable diet* OR plant-forward diet* OR vegetable-forward diet* OR meat reduction OR low meat OR low meat diet* OR plant-eating OR meat consumption OR plant consumption OR dairy consumption OR veggie OR veggie diet* OR non-dairy OR plant-derived OR sustainable food*) AND (adult* OR middle age* OR adulthood OR adult population OR young adult* OR years old OR year old*) AND (high-income country* OR high-income setting* OR HIC OR affluent country* OR america OR andorra OR antigua and barbuda OR aruba OR australia OR austria OR bahamas OR bahrain OR barbados OR belgium OR bermuda OR british virgin islands OR brunei OR brunei darussalam OR canada OR cayman islands OR channel islands OR chile OR croatia OR curacao OR cyprus OR czech republic OR denmark OR england OR estonia OR faroe

islands OR finland OR france OR french polynesia OR germany OR gibraltar OR greece OR greenland OR guam OR hong kong OR hungary OR iceland OR ireland OR isle of man OR israel OR italy OR japan OR korea OR republic of china OR republic OR korea OR kuwait OR latvia OR liechtenstein OR lithuania OR luxembourg OR macao OR malta OR monaco OR nauru OR netherlands OR new caledonia OR new zealand OR northern mariana islands OR norway OR oman OR palau OR poland OR portugal OR puerto rico OR qatar OR saint kitts and nevis OR san marino OR saudi arabia OR scotland OR seychelles OR singapore OR sint maarten OR slovakia OR slovak republic OR slovenia OR spain OR st kitts and nevis OR st martin OR states OR sweden OR switzerland OR taiwan OR trinidad and tobago OR turks and caicos islands OR united arab emirates OR united kingdom OR UK OR united states OR united states of america OR USA OR US OR uruguay OR virgin islands OR wales)

Total searches: 9665

File S5: Project student questionnaire

PROJECT STUDENT QUESTIONNAIRE

MSc: Nutrition for Global Health

Project Title: Barriers to adopting a plant-based diet: a systematic review

As part of our assessment procedure for student projects, we are asking you to complete the following short questionnaire. A copy of this questionnaire will be included in the project submission to assist the first marker's evaluation of the project.
(If filling in electronically double click on the check box to mark your choice)

Who initiated the project?

- ☐ supervisor
- ☐ co-supervisor
- ☒ student

How much help was given in developing the project?

- ☐ none: the student decided on the design alone
- ☒ some: the student used his/her initiative but was helped by suggestions from the supervisor/co-supervisor
- ☐ substantial: the supervisor/co-supervisor had most say, but the student added ideas of his/her own
- ☐ maximal: the student relied on the supervisor/co-supervisor for ideas at all stages
- ☐ not applicable: the nature of the project was such that the student had minimal opportunity to contribute to the design

How much help was given in carrying out the work for the project?

- ☐ none: the student worked alone with no supervisor/co-supervisor input
- ☐ minimal: the student worked alone with very little supervisor/co-supervisor input
- ☒ appropriate: the student asked for help when needed
- ☐ substantial: the supervisor/co-supervisor gave more assistance than expected
- ☐ excessive: the supervisor/co-supervisor had to give excessive assistance to enable the student to get data

What was the degree of technical difficulty involved?

- ☐ slight: data easily obtained
- ☒ moderate: data were moderately difficult to obtain
- ☐ substantial: data were difficult to obtain

How much help was given in the analysis and interpretation of the results?

- ☐ none
- ☒ standard: the supervisor/co-supervisor discussed the results with the student and advised on statistics and presentation
- ☐ substantial: the supervisor/co-supervisor pointed out the significance of the data and told the student how to analyse it

How much help was given in finding appropriate references?

- ☐ none

☒ some: only a few references were provided
☐ substantial: most references were given by the supervisor/co-supervisor
☐ maximal: the supervisor/co-supervisor supplied all the references used by the student

How much help was giving in writing the report?

☐ none: the supervisor/co-supervisor did not see the report until it was submitted
☐ minor: the supervisor/co-supervisor saw and commented on parts of the report
☒ standard: the supervisor/co-supervisor saw and commented on the first draft of the report
☐ substantial: the supervisor/co-supervisor gave more assistance than standard
☐ The student was at LSHTM during the writing up stage
☐ The student was out of the country during the writing up stage

How much time was spent on the project?

☐ too little to expect adequate data*
☒ sufficient
☐ too much*

**if too little or too much, were there any reasons for it, e.g. unforeseen technical problems, lack of materials, etc.?*

During the course of the work was contact with the supervisor/co-supervisor

☐ too infrequent
☐ infrequent but sufficient
☒ frequent but not excessive
☐ excessive

File S6: Data extraction form

Author, title, and year	Study geographical setting	Study population sample size, age, sex, social position	Study geographical design	Defined primary outcome	Effect measures	Key findings/ barriers	Other: funding, conflict of interest	URL

File S7: Data extraction form example

Author, title, and year	Study geographical setting	Study population sample size, age, sex, social position	Study geographical design	Defined primary outcome	Effect measures	Key findings/ barriers	Other: funding, conflict of interest	URL
Kemper. Motivations, barriers, and strategies for meat reduction at different family lifecycle stages. (2020) [40]	New Zealand	Total n=36. Age 18- 35 years. Female n=32. Males n=4.	Qualitative research with the use of focus groups interviews	Motivations	n/a	Lifestyle affected motivations and barriers perceived. Personal or family enjoyment of meat. Time and convenience as well as cost. Enjoyment of meat, beliefs and nutritional benefits were the main barriers for all to not reducing meat and moving to a plant-based diet. Meat cravings. The only way for younger individuals who enjoyed the taste was to see it as a treat and reduce it when they could. Similar to the older individuals who would try to eat meat when going out as a treat to help reduce and overcome the barrier of enjoyment/ taste. Overall, there was a big belief (from all) that meat was required to meet nutrient intake, including iron and protein. Individuals' education, knowledge and awareness would impact on meat reduction. Those with more nutritional knowledge were more likely to move towards a more plant-based diet. Transparency in the food supply chain- Once the information was known about the food chain and the consequences on the environment's health, there became a distrust for individuals towards the food supply chain and the production of their food. A counterbalance to this was for participants to check ingredient labels and make their meals at home from scratch using wholefood ingredients—a big concern with meat substitutes replacing their meat. Younger individuals were more likely to try meat substitutes compared to older individuals. Transitioning to a plant-based diet would require more energy and effort than continuing meat consumption. Things to counter these barriers included conscious meat consumption, improving health, reducing cost, helping the environment, seeing meat alternatives as adventurous, and trying new things, such as altering cooking styles, including meals and foods from different cultures, particularly spices. Reducing meat consumption was considered easier than reducing dairy and eggs. As dairy and eggs were in more food items, they craved such as baked goods. Initially, the perception of ease and time for vegetarian meals was a barrier, but after cooking these types of meals, participants agreed it was easier to make without the meat. Society, media and family pressure, and one grew up on the idea of meat and three veg. For the male participants, the idea surrounding masculinity was a major issue. This transparency affected the transition to more plant-based options as there was a concern with food processes and meat replacements.	n/a	https://www-sciencedirect-com.ez.lshtm.ac.uk/science/article/pii/S0195666319310876

File S8: Summary of individual study characteristics and findings (Full text)

Author, title & year	Study geographical setting	Study population sample size, age, sex & social position	Study design	Study outcome	Data analysis method	Key findings- Barriers
Barr and Chapman. Perceptions and practices of self-defined current vegetarian, former vegetarian, and nonvegetarian women. (2002) [38]	Canada	Total n=193. Vegetarian n=90, former vegetarian n=35, non-vegetarian n=68. Age, 18- 50-year-olds. Females, n=193. University graduate 49.7%, student 40.1%, employed 73.2%	Mixed methods. Cross-sectional survey; qualitative interviews with a subsample.	Dietary intake patterns and dietary change<br><br>Perception of meat and dairy products	Group comparisons: One-way ANOVA.<br><br>Continuous variables: Post-hoc testing<br><br>Categorical variables: Chi-squared	Results from all groups, with group differences, with means from a 5-point Likert scale, one strongly disagrees to five which strongly agree:<br>Enjoying the flavour of red meat. Vegetarians 10% of participants agree or strongly agree (M=1.6, SD=1.1); former vegetarians 40% of participants agree or strongly agree (M=3.0, SD=1.3); nonvegetarians 59% of participants agree or strongly agree (M=3.5, SD=1.0). A statistically significant difference between groups by analysis of variance (p<0.001)<br><br>Red meat is part of a healthy diet. 41% of Vegetarians agree or strongly agree (M=2.8, SD=1.2), 66% of former vegetarians agree or strongly agree (M=3.7, SD=0.9), 85% of nonvegetarians agree or strongly agree with the statement (M=4.0, SD=0.9). A statistically significant difference between groups by analysis of variance (p<0.001)<br><br>Diets that contain red meat are healthier than those that do not. 6% of vegetarians (M=1.5, SD=0.9), 12% of former vegetarians (M=2.3, SD=0.9), and 25% of nonvegetarians agree or strongly agree with the statement (M=2.7, SD=1.0). A statistically significant difference between groups by analysis of variance (p<0.001)<br><br>Dairy products provide essential protein and nutrients. 60% of vegetarians agree or strongly agree (M=3.3, SD=1.3), 71% of former vegetarians (M=3.9, SD=1.0), and 79% of nonvegetarians agree or strongly agree with the statement (M=4.0, SD=1.1). A statistically significant difference between groups by analysis of variance (p<0.001)<br><br>Results from non-vegetarians who used to be a vegetarian:<br>Health-related issues include concerns for weakness, fatigue and anaemia (n=10).<br><br>Missing the taste of meat and enjoyment of eating meat (n=8).<br><br>Changes in living situations, including moving back to their family home where the family were meat-eaters (n=7).<br><br>Other health-related issues surrounding nutritional concerns. This included the reference with not getting enough protein in their diets (n=5)—worries regarding other nutrients such as B12, calcium, and iron.<br><br>Consuming a vegetarian diet would require too much time to prepare, cook, and follow (n=6).<br><br>There was a lack of social support; if family members they lived with or friends were non-vegetarians/ meat eaters, it was harder to follow a plant-based diet (PBD).

Author, title & year	Study geographical setting	Study population sample size, age, sex & social position	Study design	Study outcome	Data analysis method	Key findings- Barriers
Faber, Castellanos-Feijoo, Van de Sompel, Davydova and Perez-Cueto. Attitudes and knowledge towards plant-based diets of young adults across four European countries. Exploratory survey. (2020) [39]	Belgium, Denmark, Netherlands and Spain	Total n=438, Belgium n=110, Denmark n=119, Netherlands n=116, Spain n=93. Age, 18- 30 years. Females 56- 82% across countries. Males 18- 44% overall across countries.	Quantitative cross-sectional study	Awareness of plant-based diets- perception and attitudes<br><br>Appeal of diet terminology	Differences among countries: Pearson's Chi-squared test and Bonferroni.<br><br>Proportions: Fishers exact test<br><br>Continuous variables: Kruskal-Wallis test<br><br>To test the association between knowledge and attitudes towards plant-based (PB), vegan, and vegetarian diet terms. Adjusting for age, sex, etc: Logical regression	Participants had limited knowledge of PBD composition; many believed it was the same as following a vegan diet. Awareness of the term PBDs varied significantly per country when adjusted for multiple group comparisons using a Bonferroni test (P<0.001). Mean values and interquartile ranges were coded against a 5-point Likert scale. Value one being totally disagree, and value five being totally agree. Belgium (M=2.0, IQR=2.0-3.0), Denmark (M=3.0, IQR=2.0-4.0), Spain (M=2.0, IQR=1.0-4.0), the Netherlands (M=3.0, IQR=2.0-4.0). Difficult concepts to understand due to the numerous terminologies surrounding the topic.<br><br>Participants from Spain were the least aware of PBDs (38%) compared with Denmark, where awareness was 83%. However, despite a lack of awareness in Spain, participants expressed a more positive attitude toward PBDs (M=3.5, SD=0.68, P=0.012) than in other countries.<br><br>In Belgium and the Netherlands, there was a significant negative feeling concerning the convenience of a PBD (P<0.001) compared to Spain. Belgium (M=2.0, IQR=2.0- 3.0), Netherlands (M=2.0 IQR=2.0- 3.3), Spain (M=3.0 IQR=3.0- 4.0).<br><br>Those who perceived a PBD as tasty had 1.53 (95% CI 1.16,2.03, p=0.003) times the odds of believing the term PBDs would be more appealing than a vegetarian diet. Those who perceived a PBD as enjoyable had 1.70 (95% CI 1.31,2.24, p<0.001) times the odds of believing the term PBDs more appealing than a vegetarian one. PBDs were considered more appealing by taste and enjoyability than a vegan diet (OR=1.33, 95% CI 1.06,1.69, p=0.016, OR=1.29, 95% CI 1.04,1.60, p=0.021, respectively).
Kemper. Motivations, barriers, and strategies for meat reduction at different family lifecycle stages. (2020) [40]	New Zealand	Total n=36. Age 18- 60 years. Female n=32. Males n=4.	Qualitative research with the use of focus groups interviews	Motivations<br><br>Barriers<br><br>Strategies<br><br>Meat reduction	Thematic analysis	Barriers to meat reduction are similar across all family life cycle stages.<br><br>The main barrier for all ages was the belief around the nutritional benefits of consuming meat. Meat meets nutritional requirements, such as iron and protein.<br><br>Education, knowledge and awareness surrounding meat reduction would impact meat intake. Those with more nutritional knowledge were likelier to move towards a more PBD.<br><br>Personal or family enjoyment of meat.<br><br>Time and convenience of creating plant-based meals.<br><br>Cost of reducing meat and including plant-based foods.<br><br>Meat enjoyment and the cravings for meat.<br><br>The younger participants who enjoyed the taste of meat would try to see it as a treat to reduce their intake when possible.

Author, title & year	Study geographical setting	Study population sample size, age, sex & social position	Study design	Study outcome	Data analysis method	Key findings- Barriers
						The older participants would also try to see meat as a treat and only consume it when going out to eat to help reduce intake. Seeing meat as a treat helped participants overcome this meat enjoyment and cravings barrier.<br><br>Transparency in the food supply chain. Participants felt distrust towards the food supply chain and the understanding of how their food was produced. This transparency affected the transition to more PB options as there was a concern with food processes, particularly around meat replacements. Individuals would check ingredient labels to overcome this barrier and make their meals at home from scratch using whole food ingredients.<br><br>Family pressures and expectations to follow a meat diet and not a PBD.<br><br>For male participants, the idea of masculinity surrounded the perception of needing to consume and cook meat.<br><br>Social media as participants grew up on advertisements that promoted the idea of "meat and three veg".<br><br>Meat substitutes. Concerns surrounding meat substitutes as replacements for the reduced meat intake. Younger participants were more willing to try meat replacements when compared to the older participants.<br><br>The energy and effort required to transition to a PBD were too much for individuals compared to continuing to eat meat.<br><br>Reducing meat was deemed easier than reducing or removing other non-plant-based foods such as dairy and eggs. Both foods were considered to be in more food items, such as baked goods. Therefore, harder to avoid.<br><br>Conscious meat consumption was seen as a way to counteract these barriers. Facilitators such as improving health, reducing cost, helping the environment, seeing meat alternatives as adventurous or seeing it as trying a new thing, trying to cook in a new way with different and new ingredients such as spices.<br><br>The perception of the difficulty and time required to prepare vegetarian meals was a barrier. However, participants agreed it was easier to cook without the meat after learning to cook vegetarian meals.

Author, title & year	Study geographical setting	Study population sample size, age, sex & social position	Study design	Study outcome	Data analysis method	Key findings- Barriers
Kemper and White. Young adults' experiences with flexitarianism: The 4Cs. (2021) [41]	New Zealand	Total n=23. Age 18-35 years. Female n=17. Males n=6. Students, 100%	Qualitative research with an exploratory approach using semi-structured interviews	Lived experiences<br><br>Motivations<br><br>Strategies<br><br>Barriers<br><br>Meat reduction and transition to a flexitarian diet	Thematic analysis	Not having control or autonomy over meals reduced participants from adopting a more flexitarian diet. But on the other hand, moving away from home allowed control and opportunities to regulate meat consumption.<br><br>Overall cooking ability and confidence. In particular, lacking confidence in cooking with plant-based foods. These include lentils, legumes, and tofu.<br><br>Not having access or opportunities to buy groceries. However, those who could buy their food were allowed to improve their knowledge and exploration of plant-based foods.<br><br>Time-consuming especially seemed challenging to adopt a more flexitarian diet with busy schedules.<br><br>Nutritional concerns and dietary diversity.<br><br>Cravings and taste.<br><br>Bodily reactions such as fullness and satisfaction played a key role in not wanting to reduce meat consumption. Particularly after physical activity, cravings for meat were higher.<br><br>Accessibility/ lack of meat-free options when eating out.<br><br>Social aspects such as not wanting others to feel they were enforcing their beliefs about adopting a more flexitarian diet onto others. They also didn’t want to make a fuss or have to justify their eating habits to others in social situations.<br><br>Speaking to family and friends about a flexitarian diet was more positively received than saying a meat-free, vegetarian or vegan diet. Living with similarly minded individuals when accepting and trying new things, such as reducing the meat diet, increased the chance of moving towards a PBD.<br><br>Participants felt that being either a meat-eater or vegetarian was a barrier. Compromise played an essential role in moving towards a more PBD. Not having to classify themselves and comprise at times made transitioning to a more PBD more manageable.<br><br>Dairy and eggs were the hardest to find the motivation to reduce or cut out their diet. Milk, however, was the easiest to reduce. PB milk substitutes were the most accessible, readily available, tasty, and affordable compared to cheese and egg replacements/ PB options.<br><br>Spreading meat across the week and including PBFs such as pulses and vegetables in combination with meat helped reduce overall meat consumption.<br><br>Concern for the environment increased the chance of adopting a more PBD.

Author, title & year	Study geographical setting	Study population sample size, age, sex & social position	Study design	Study outcome	Data analysis method	Key findings- Barriers
Lea, Crawford and Worsley. Public views of the benefits and barriers to the consumption of a plant-based diet. (2006) [42]	Australia	Total n=415. Ages 20- 65 years. Female 59.4%. Male 40.6%. Employed full time, 31.5%, employed part-time, 17.8%, unemployed 2%.	Quantitative cross-sectional survey study design	Perception and attitudes towards dietary intake/ change<br><br>Barriers<br><br>Benefits	Frequency of responses: Pearson's Chi-squared test. Adjusted by sex, age, and university education.	There was a lack of perceived information regarding PBDs. 42% of participants agreed that this was the most significant barrier.<br><br>Lack of autonomy over diet.<br><br>Unwillingness or inability of themselves or family members to change their dietary patterns.<br><br>Lack of availability of PB options when trying to eat out.<br><br>A belief that PBDs were not tasty.<br><br>Health and nutritional concerns were not deemed as a barrier.<br><br>Differences were seen between participants as non-university educated and older participants were less willing to change their diets than those younger and went to university. Women were less likely than men to believe humans needed or were meant to eat meat, particularly in large quantities.<br><br>However, there were perceived health benefits of adopting a PBD, which included a decrease in saturated fat (79% agreed), helping to prevent diseases (70%) and increased fibre intake (76%).
Lim, Okine and Kershaw. Health- or environment -focused text messages as a potential strategy to increase plant-based eating among young adults: an exploratory study. (2021) [43]	United States of America	Total n=159. Ages 18- 26 years. Females n=107. Males n=49 Other n=3.	Quantitative 8-week text message pre-and post-intervention	Dietary beliefs, intentions, behaviour and intake	Participant's characteristics group comparisons: Chi-squared test.<br><br>Explore the effect of the interventions on dietary predictors, intentions and behaviours: Paired samples t-test, from baseline and post-intervention answers.<br><br>Differences between group intervention: Independent samples t-test.	Food neophobia played an important role in transitioning to eating Plant-based foods. Individuals may be reluctant to try new foods, which becomes a barrier to introducing plant-based foods and adopting this diet. No significant association between interventions and the introduction of plant-based foods such as plant protein. Mean value differences in changes in dietary predictors, intentions, intakes, and intentions after the text message interventions for each intervention group. Health messages (MD=0.20, p=0.400). Environment messages (MD=0.05, p=0.811).<br><br>A lack of familiarity with plant-based food, particularly PB proteins, was a barrier.<br><br>However, the individuals who received the environment-focused text message intervention had a greater change in dietary beliefs overall. In relation to their values and perceived benefits of a PBD, as seen with mean differences from before the intervention to after in each intervention group (MD=0.25, p=0.007), (MD=0.27, p=0.011) respectively. They were shown to be more likely to increase vegetable intake (MD=0.35, p=0.015).<br><br>The strongest predictors of having the intention to reduce the consumption of animal foods compared to plant foods were self-efficacy (r=0.081, B=0.175, p=0.018), subjective norms (r=0.037, B=0.148, p=0.036) and moral satisfaction (r=0.219, B=0.181, p=0.007).

Author, title & year	Study geographical setting	Study population sample size, age, sex & social position	Study design	Study outcome	Data analysis method	Key findings- Barriers
Macdiarmid, Douglas and Campbell. Eating like there's no tomorrow: public awareness of the environmental impact of food and reluctance to eat less meat as part of a sustainable diet. (2016) [44]	Scotland	Total n=83. Ages 25- 56 years. Females n=43. Males n=40.	Qualitative research with the use of focus groups interviews	Public awareness<br><br>Public willingness to make dietary changes.<br><br>Meat reduction	Thematic analysis<br><br>Exploring deprivation, sex or urban/ rural differences in attitudes towards reducing meat consumption: Framework analysis.	Scepticism around the benefits of adopting a PBD, particularly for the environment. Believe that consuming meat was trivial. Individuals didn't consider any link between food consumption and environmental impact.<br><br>A lack of awareness around how individuals' eating habits and food production impact climate change. Believing changing their diets had little to no difference on the environment.<br><br>Meat is pleasurable.<br><br>Social roles and traditions played a key role in preventing meat reduction.<br><br>A strong sense of powerlessness and mistrust of the food supply industry. The perceived idea is that locally-grown organic food is preferable and more trustworthy.<br><br>Even those aware of their diet's impact on the environment still believed the valuable nutrient content of non-plant-based foods was essential for human health.<br><br>Believed despite any benefits of adopting a PBD, tradition was more important. Meat had to be included in a meal for it to be considered a proper meal.<br><br>Distrust in the advice given by individuals, particularly health professionals, as advice is forever changing.<br><br>The idea is that other countries are having a worse impact on the planet than they are as individuals.<br><br>Some believed they wouldn't need to reduce further if they had already eaten a small amount of non-plant-based foods.<br><br>No consensus on the definition of meat, which could cause some confusion. Some believed meat was red meat only. Others said real meat was, excluding sausages, burgers, etc.

**Author, title & year**	**Study geographical setting**	**Study population sample size, age, sex & social position**	**Study design**	**Study outcome**	**Data analysis method**	**Key findings- Barriers**
Makiniemi and Vainio. Barriers to climate-friendly food choices among young adults in Finland. (2014) [45]	Finland	Total n=350. Mean age 24. Females n=280. Males n=70. Students 100%	Quantitative cross-sectional questionnaire study	Perception and attitudes, and barriers toward dietary intake/ change	Differences in men and women for perceived barriers: t-test.<br><br>The impact of perceived barriers on climate friendly food choices: Multiple regression analysis.	The perceived high price of climate-friendly foods was perceived as the most relevant and important barrier (r=.31, SD=1.50, p=<0.001).<br><br>Poor supply and accessibility of climate-friendly foods (r=.19, SD=1.32, p=<0.001).<br><br>A lack of knowledge surrounding climate-friendly food choices and, therefore, difficulty in making these choices (r=.27, SD=1.50, p=<0.001), (r=.64, SD=1.40, p=<0.001), respectively.<br><br>Lack of time (r=.26, SD=1.44, p=<0.001).<br><br>There is a fear of missing out and wanting to eat the same foods as others (r=.20, SD=1.59, p<0.05).<br><br>Compared to men, women perceived high prices of climate-friendly foods as a more important barrier. As well as a poor supply of these foods (t=2.29, SD=1.47, p<0.05). In contrast, men perceived disbelief in food choice and its impact on the climate and wanted to have the same habitual diet as more important than women (t=-2.78, SD=1.50, p<0.001), (t=-3.2, SD=1.81, p<0.001) respectively.<br><br>Those already consuming a vegetarian diet perceived the barriers as less important than non-vegetarians. For example, high prices (t=-3.90, SD=1.61, p<0.001) compared to non-vegetarian (t=-3.90, SD=1.44, p<0.001).
Markowski and Roxburgh. "If I became a vegan, my family and friends would hate me:" Anticipating vegan stigma as a barrier to plant-based diets. (2019) [46]	United States of America	Total n=34. Females n=26. Males n=8. Students 100%.	Qualitative interviews with the use of focus groups	Individual perceptions<br><br>Stigmatisation<br><br>Barriers<br><br>Meat consumption	Coding of transcripts	Individuals who were vegan responded positively to descriptors related to vegans and veganism.<br><br>Non-vegans viewed vegans and veganism negatively. For example, words such as “crazy” or “stupid” were associated with non-vegans.<br><br>Negativity with vegan eating patterns being deemed as abnormal or irrational.<br><br>Socially adopting a vegan diet could be a problem. Vegetarians viewed vegans as attention seekers who act morally superior.<br><br>Not being able to eat in social situations. Having to refuse food might be seen as socially unacceptable.<br><br>Violating food norms by eating non-animal products.<br><br>Less stigma with vegetarians compared to vegans.<br><br>Omnivores would physically and verbally distance themselves from vegans due to perceived stigma. Vegetarians were less concerned with physical distancing.

Author, title & year	Study geographical setting	Study population sample size, age, sex & social position	Study design	Study outcome	Data analysis method	Key findings- Barriers
						Anticipating stigma. Such as how others will treat them due to their new diet.<br><br>Stigma, in particular from family members.<br><br>Resisting the temptation of food that's non-plant-based.<br><br>Knowing what foods to eat instead of non-plant-based foods.<br><br>Knowledge of how to prepare non-animal products.<br><br>Learning to ignore negative comments from adopting a PBD.<br><br>Negative social repercussions of going vegan.<br><br>Having a support network or supportive family could help them move towards/ reduce these barriers.
Von Essen. Young adults' transition to a plant-based diet as a psychosomatic process: A psychoanalytically informed perspective. (2021) [47]	Sweden	Total n=9. Ages 18-35 years.	Qualitative semi-structured interviews	Individual perceptions<br><br>Dietary transition<br><br>Challenges	Descriptive phenomenological psychological method	Exploring new ways of living based on health anxieties. Anxiety with being unable to obtain all the nutrients needed from a PBD.<br><br>Having no autonomy with food choices.

File S9: Quality appraisal example

Study: Barr and Chapman. Perceptions and practices of self-defined current vegetarian, former vegetarian, and nonvegetarian women. (2002)		
**Appraisal indicator**	**Result**	**Comments**
Did the study address a clearly focused question/issue?	Yes	The study had a clear question that they wanted to address. This was to assess the dietary practices of vegetarians and how this may have differed over time. As well as the aim to explore perceptions of meat and dairy for different groups of vegetarians, former vegetarians and non-vegetarians. A rationale is also provided for why the question needs to be addressed.
Is the research method (study design) appropriate for answering the research question?	Yes	A cross-sectional survey was applied as the data collection method. This included demographic information, perceptions, and attitudes on aspects of food habitats over time. The survey design was pretested to allow for any important but minor modifications. A subsample of 15 participants who were former vegetarians was also conducted. This combination for the study design gained the information needed to answer the research question regarding dietary practices but also allowed for in-depth data to be collected as to why the former vegetarian resumed an omnivore diet.
Is the method of selection of the subjects (employees, teams, divisions, organisations) clearly described?	Yes	Convenience sampling was used to recruit premenopausal women who were either vegetarian, former or non. This means participants were aged 18 to 50 years. The method of recruitment was done through notices in universities and community newspapers. Word of mouth was also used due to the sampling method. The study then goes into further detail about the selection of participants for the subsample and the requirements for this component selection.
Could the way the sample was obtained introduce (selection) bias?	Yes	As the sampling was convenience and the use of word of mouth was used to obtain certain participants, this would most likely introduce selection bias. This study may then also lack generalisability.
Was the sample of the subject's representative with regard to the population to which the finding will be referred?	Yes	The sample of participants was representative of the study population to which the findings were referred. The participants were women aged 18- 50 who followed one of the three diets. Various dietary practices were considered, which helped reflect the study population that the findings would apply. This was made clear in the researcher's study.
Was the sample size based on pre-study considerations of statistical power?	No	There was no information regarding statistical power to obtain the required sample size. The only aspect to this stated was using 15 participants for the subsample. The rationale was that in-depth qualitative interview studies typically use this sample size. Therefore, there was either not enough information or justification for this indicator.
Was a satisfactory response rate achieved?	Can't tell	One hundred ninety-three participants completed the survey. However, it's difficult to understand if a satisfactory response rate was achieved as it's unclear how many individuals received but did not complete/ participate in the survey.
Are the measurements (questionnaire) likely to be valid and reliable?	Yes	The survey is likely valid as it measures perceptions of meat and dairy products and other dietary behaviours. This was done on a 5-point Likert response scale. However, reliability may be an issue. The first part of the survey would be reliable. Still, within the survey, there was an open-ended question asking former vegetarians about what made them decide to resume an omnivore diet. Therefore, the measurement for this section would not be repeatable due to its open-ended nature. The survey was also pretested by representative participants of the study. Overall, from all the information presented in the study, I would say the measurement was valid but might lack some reliability.
Was the statistical significance assessed?	Yes	A one-way variance analysis was conducted using post hoc comparisons (Scheffes test) to test for differences between vegetarians, former and non. Then, a chi-squared test for differences was also conducted. Finally, statistical significance was tested and stated.
Are confidence intervals given for the main results?	No	No confidence intervals are provided, only means and standard deviation plus group differences and statistical levels.
Could there be confounding factors that haven't been accounted for?	Can't tell	Confounding factors were not expressed in the study. None were accounted for.
Can the results be applied to your organisation?	n/a	n/a
**Overall score: 6 out of 11**	**Overall rating/ grading: moderate**	

File S10: Risk of bias assessment results in detail.

Qualitative studies

The qualitative studies returned positive results for the first four indicators. However, all studies did not address whether the researcher's and participants' relationships were adequately considered. This could alter the results depending on how familiar the researcher and participants are/ what their relationship is—creating acquiescence bias. All studies addressed ethical issues, obtained sufficient ethical approval, and provided adequate information regarding potential ethical issues such as confidentiality. The final indicator was met by all but two studies. The first of the two studies [46] did not meet this indicator as the valuableness of the study findings was related to an overall reduction in meat consumption and not the results of the actual study. The second study's [47] findings were not transferrable to other study populations, and real-world implications were not stated. This study may, therefore, lack external validity.

Quantitative studies

Three out of the four studies had issues with their selection of participants. The sampling methods ranged from snowball to convenience sampling. Most of these participants were not randomly selected. In one study using data from Belgium, Denmark, the Netherlands and Spain [39], researchers recruited participants in their network via their email/ WhatsApp messages. Therefore, in the quantitative studies, selection bias was a clear source of bias. This bias would affect the validity of the studies as this was concerning participant selection. This could lead to issues with external validity. Only this one study [39] used a sample size based on pre-study-considerations-of-statistical power. Therefore, the other four studies may have inadequate participants to obtain statistical power, reducing the chances of detecting a true effect. Overall, many studies (three out of four) did not include confidence intervals in their main results alongside effect measures. This could affect the interpretation and conclusions made for each study result. Confounding was not a risk factor in all but one study. The researchers addressed confounding during the data analysis phase through adjustments, multiple regression analysis, etc. It was undetermined for the one Canadian study as these needed to be explicitly addressed or accounted for [38].

For the pre-and post-intervention study set in the USA [43], 13 of the 27 indicators showed that the study was conducted/ designed to minimise the risk of bias. However, three aspects of the checklist were not reported; therefore, it was impossible to make assumptions about these. These could be a potential source of bias. This included allocation of concealment/ blinding and balancing the overall harm and benefits of the intervention. Due to the nature of the study, concealment/ blinding was most likely not applicable. Lastly, the study population/ area used needed to be representative of the source. The age of participants was narrow (18- 26). The study also needed to explain where the source population was from in the USA, whether this was a rural or urban setting. This may reduce the external validity of the study results. In addition, there could have been issues with the outcome measures as these were subjective as the information was self-reported. This could cause response bias and social desirability bias, for example. Finally, confidence intervals were not stated. However, p values were given as part of the main results. This could lead to inaccuracies in the findings, as the magnitude of the effect and statistical plausibility may not be a reliable estimate.

File S11: Certainty assessment results

Barriers identified	Level of Certainty	Times mentioned
1. Perception of high prices for plant-based foods	Moderate	39, 44
2. Cost	Low	39
3. Lack of information on knowing what to eat	Moderate	38, 39, 41, 44, 45
4. Lack of familiarity with plant-based foods/ diets	Moderate	38, 39, 42, 45
5. Nutritional knowledge	Moderate	37, 39, 43, 46
6. Believing eating patterns are trivial	Low	43
7. Education	Moderate	39, 41
8. Confusion surrounding the term and definitions of plant-based diets	Moderate	38, 40, 43, 45
9. Distrust/mistrust in food systems and suppliers	Moderate	39, 43, 44
10. Stigma	Moderate	38, 40, 45
11. Food neophobia	Low	41
12. Gender stereotypes	Moderate	39, 41
13. Fear of judgement	Moderate	40, 45
14. Powerlessness over food choices	Moderate	40, 43, 44, 46
15. Nutritional deficiencies	Moderate	37, 41, 46
16. Nutritional intake/ requirements	High	37, 39, 40, 41, 45, 46
17. Dietary diversity	Moderate	37, 40
18. Anxiety with the worry of not consuming the necessary nutrients	Moderate	37, 46
19. Time	Moderate	37, 39, 40, 44
20. Food preparation	Moderate	37, 39
21. More energy and effort needed	Low	39
22. Difficulties with creating plant-based meals	Moderate	37, 39
23. Fear of missing out/ not following social norms	Moderate	40, 44, 45
24. Lack of support/ a support network	Moderate	37, 45
25. Living situation	Moderate	37, 40
26. Traditions	Low	43
27. Family and friend expectations and pressures	Moderate	37, 39, 45
28. Personal and family enjoyment	Moderate	37, 38, 39
29. Resisting temptations	Low	45
30. Cravings	Moderate	39, 40, 43
31. Taste	Moderate	37, 40, 41
32. Scepticisms around the dietary impact	Moderate	43, 44
33. Disbelief in climate change	Low	43
34. Lack of options, especially when eating out	Moderate	40, 43, 45
35. Lack of plant-based options to buy	Moderate	40, 44
36. No autonomy/ opportunity over food purchases	Moderate	37, 40, 41, 46

Barriers identified	Level of Certainty	Times mentioned
37. Lack of confidence in cooking ability and preparing plant-based meals	Low	40
38. Cooking ability	Moderate	37, 40
39. Habit	Moderate	39, 41, 43, 44, 46
40. Advertisements promoting meat consumption	Low	39

Figure 4. Certainty assessment results. The first column shows the 40 barriers identified. The second column is the level of certainty. This ranged from low, moderate to high. A low level of certainty is when one study mentioned that barrier. A moderate level of certainty ranged from 2 to 5 studies mentioning that particular barrier. Lastly, a high level of certainty included six or more studies showing evidence that a particular barrier prevented individuals from adopting a PBD. Every barrier was mentioned at least once; the most a barrier was mentioned was six times.
